# Honeydew microbial ecology: a neglected frontier in multitrophic networks

**DOI:** 10.1093/femsec/fiag104

**Published:** 2026-09-08

**Authors:** Francine A C van Neerbos, Antonino Cusumano, Bart Lievens, Maite Fernández de Bobadilla

**Affiliations:** Institute of Ecology, University of Bremen, D-28359, Bremen, Germany; Department of Agricultural, Food and Forest Sciences, University of Palermo, I-90128, Palermo, Italy; CMPG Laboratory of Process Microbial Ecology and Bioinspirational Management, Department of Microbial and Molecular Systems, KU Leuven, B-3001, Leuven, Belgium; Leuven Plant Institute (LPI), KU Leuven, B-3001, Leuven, Belgium; Centro de Protección Vegetal, Instituto Valenciano de Investigaciones Agrarias (IVIA), Moncada, 46113 Valencia, Spain

**Keywords:** ecology, entomology, honeydew, insect–microbe interactions, microbiology, multitrophic interactions

## Abstract

Honeydew, the sugary excretion produced by sap-feeding Hemiptera, is one of the most common carbohydrate resources in many plant-based food webs. Honeydew supports a wide range of organisms, including ants, pollinators, (hyper-)parasitoids wasps, predatory insects, and microbes. As a sugar-rich resource, honeydew is frequently colonized by specific microbes (i.e. bacteria and fungi), which consume its sugars and other nutritional constituents. Recent research suggests that microbes within honeydew may modify its traits. Thereby driving microbial succession and acting as important ‘hidden players’ in multitrophic ecological interactions. Yet, its role as a dynamic microbial habitat remains largely unexplored. Here, we synthesize current evidence on the microbial ecology of honeydew and propose that honeydew is a dynamic rapidly changing microbial habitat. We further propose a four-stage successional model to frame its temporal dynamics. We discuss how the honeydew microbiome alters nutritional composition. Further, we discuss how the honeydew microbiome may mediate multitrophic interactions through the emission of volatile organic compounds that attract natural enemies of honeydew-producing insects. We propose that this process may pose a trade-off between the microbial secondary metabolism and its dispersal capacity of some microbes. We aim to stimulate research that will establish honeydew microbial ecology as a new frontier in plant–insect–microbe interactions.

## Teaser

Review and synthesis of the microbiology, ecology, and microbial ecology of honeydew, the sugary excretion of sap-feeding hemiptera.

## Introduction

Honeydew, the sugar-rich excretion of sap-sucking Hemiptera, represents one of the most abundant and spatially widespread sugar resources in terrestrial ecosystems (Douglas 2009). By feeding on phloem sap, insects like aphids, whiteflies, mealybugs, psyllids, and soft scales ingest large volumes of sugar-rich fluid and excrete excess carbohydrates as honeydew. In addition to carbohydrates, honeydew contains, in smaller amounts, amino acids, organic acids, minerals, and reactive oxygen species (ROS) derived both from plant sap and insect metabolism (Ewart and Metcalf 1956, Tena et al. 2013, Urbaneja-Bernat et al. 2024). Because it is produced by numerous hemipteran species and is often available year-round, honeydew forms a major nutritional input of plant-based food webs and supports diverse organisms (Fernández de Bobadilla et al. 2024), including ants (Fischer and Shingleton 2001), predators and parasitoids of herbivores (reviewed in Wäckers et al. 2008), hyperparasitoids (i.e. secondary parasitoids that develop on or within parasitoids) (van Neerbos et al. 2020), pollinators (Jones and Rader 2022), reptiles (Fölling et al. 2001), birds (Nascimento de Mattos et al. 2025), primates (Joly-Radko and Zimmerman 2010), and microbes (Leroy et al. 2011, Hung et al. 2015, Glacet et al. 2025, Liu et al. 2025).

Upon excretion, honeydew is rapidly colonized by microorganisms, mainly originating from the honeydew-producing insect gut and the plant phyllosphere (Leroy et al. 2011, Álvarez-Pérez et al. 2024). Fermentative yeasts and bacteria then quickly modify its chemical and nutritional composition as well as its detectability by generating volatile organic compounds (VOCs) (Glacet et al. 2025, Li et al. 2025, Liu et al. 2025). As in other ephemeral, sugar-rich substrates (e.g. (extra-) floral nectar and rotting fruits), these microbial transformations occur over short timescales and can influence interactions across multiple trophic levels (Butterworth et al. 2022). For instance, microbial fermentation can reduce attraction and nutritional suitability for some consumer insects, while enhancing attraction for others (Goelen et al. 2018, 2021, van Neerbos et al. 2023a, b, Zhang et al. 2023, Li et al. 2025, Liu et al. 2025). Bacterial volatiles associated with sugar-rich substrates can elicit contrasting behavioural responses across trophic levels (van Neerbos et al. 2023a). Specifically, bacterial volatiles, depending on the VOC composition and concentration, can simultaneously attract particular insects such as aphids and parasitoids while deterring others such as hyperparasitoids (van Neerbos et al. 2023a). As a result, the microbial ecology of honeydew potentially reshapes ecological networks by altering resource quality, consumer behaviour, and species interactions.

Despite honeydew’s central role in multitrophic networks and increasing evidence that microorganisms rapidly transform it, the microbial ecology of honeydew remains poorly understood. Research on the microbial ecology of floral nectar, another ephemeral plant-derived sugary secretion, has demonstrated how microbial dispersal ability, priority effects (i.e. the influence of early-arriving microbes on the establishment and function of later colonists), ecological filtering (i.e. selective constraints imposed by the nectar environment, mainly due to its high osmotic pressure), species interactions (e.g. competition and facilitation), and microbial activity (e.g. VOC production and reduction of secondary plant metabolites) can drastically alter plant–insect interactions (Vannette and Fukami 2016, Sobhy et al. 2018), Cusumano et al. 2023, Ermio et al. 2024, Quevedo-Caraballo et al. 2024, Chappell et al. 2025). Likewise, similar predictable successional dynamics are well described for spontaneously fermented traditional foods and beverages, such as cocoa beans and lambic beer (De Roos and De Vuyst 2018, De Vuyst and Leroy 2020).

Microbial communities in carbohydrate-rich substrates often follow distinct, time-structured trajectories. This suggests that honeydew, too, is likely to be subject to similar principles and undergo its own characteristic microbial succession. Yet, we currently lack a conceptual framework to understand how the honeydew microbiome assembles, functions, changes over time, and mediates ecological interactions. Here, we propose such a framework by drawing parallels with the microbial interactions within nectar and spontaneously sugar-rich fermented food and beverage microbiomes. We aim to position honeydew as a key microbial niche mediating species interactions.

In this manuscript, we:

1. Integrate current knowledge on the microbiology of honeydew and its effects on its chemistry and its volatilome (total set of VOCs).
2. Propose a four-stage successional hypothetical framework describing microbial assembly in honeydew, shaped by ecological filtering, priority effects, and chemical modifications; and
3. Link these dynamics to cascading effects on multitrophic interactions, with implications for ecosystem functioning and biological control.

By identifying key knowledge gaps and proposing testable hypotheses, we aim to establish honeydew microbial ecology as a new frontier in the study of plant–microbe–insect interactions.

## The microbiome of honeydew

Historically, honeydew microbial research primarily focused on sooty moulds, particularly due to their negative economic impact (Chomnunti et al. 2014, Flessa et al. 2021). More recently, studies have started to investigate the bacterial and fungal diversity within honeydew beyond sooty moulds (Vasse et al. 2017, Cheng et al. 2024, Li et al. 2025, Liu et al. 2025, Zhao et al. 2026). However, integrated research where both the bacterial and fungal diversity, as well as their dynamics, are investigated is lacking (Flessa et al. 2021, Glacet et al. 2025). Furthermore, based off of the absence of reports during the preparation of our review, we found no reports which currently focused on either the viral or archaeal diversity present within insect honeydew.

Honeydew microbial colonizers need to overcome several physiological challenges that differ from those in the phyllosphere (Lievens et al. 2014). Microbes colonizing honeydew must cope with a high carbon-to-nitrogen ratio, high osmotic pressure, locally limited oxygen availability, the presence of plant metabolites, and the occurrence of ROS (Table 1) (Lievens et al. 2014, Calvo-Agudo et al. 2020, Urbaneja-Bernat et al. 2024). Further, honeydew is ephemeral in nature due to its depletion by consumers, washing away by rain and microbial degradation (Butterworth et al. 2022, Glacet et al. 2025).

**Table 1:** Key characteristics of insect honeydew.

Property	Range	Identity	Note	References
Bacterial cell counts	10^4^–10^7^ CFU/ml	NA	Cell counts have only been published for specific bacterial species, not for total culturable cell content. Further, cell counts depend on honeydew age.	Leroy et al. (2011), Nadarasah and Stavrinides 2011), Rodrigues et al. (2006)
Osmotic pressure	0.95–1.2 MPa	NA	Depends on the presence of endosymbionts within the aphid, as well as the carbohydrate composition and content of the ingested food. Further depends on presence of osmoregulation genes within the honeydew producing insect.	Douglas (2006), Wilkinson et al. (1997), Wintraube et al. (2025).
pH	3.0–8.0	NA	Depends on insect species, plant species, and microbial colonization of honeydew	Auclair et al. (1982), Ashbolt and Inkerman (1990), Garcia et al. (2007)
Water level	20%–30% w/v	NA	Depends on insect species, plant species, geographical region, and environmental conditions	Hijaz et al. (2015)
Viscosity	NA	NA	Only qualitative descriptions available, described as ‘sticky’	Faria et al. (2008), Urbaneja-Bernat et al. (2024)
Carbohydrate content	15–40 g/l	Glucose, fructose, sucrose, melezitose, maltose, raffinose, trehalose, and xylose	Depends on insect species, plant species, insect age, microbial colonization, presence of endosymbionts in the insect, geographical region, and environmental conditions (i.e. relative humidity, temperature, etc.)	Blanchard et al. (2022), Fokkema et al. (1983), Gray (1952), Hogervorst et al. (2008), van Neerbos et al. (2020), Wilkinson et al. (1997)
Amino acid content	2%–10% w/v	Alanine, arginine, asparagine, aspartic acid, cysteine, glutamine, glycine, glutamic acid, histidine, isoleucine, leucine, lysine, phenylalanine, proline, serine, tryptophan, tyrosine, and valine	Nonexhaustive list of amino acids detected in honeydew. Dependent on plant species, ant-tending, and environmental conditions.	Pringle et al. (2014), Yao and Akimoto (2002)
Organic acid content	2%–5% w/v	Arachnidate, behenoate, benzoate, caproate, citrate, citric acid, fumarate, glycerate, hydroxybenzoate, isocitrate, itacontate, lactate, levulinate, malate, malonate, oleate, oxalate, oxaloacetate, oxyvalerate, palmitate, stearate, succinate, succinic acid, and vanillate	Nonexhaustive list of organic acids detected in honeydew. Dependent on the insect species and plant species.	Dhami et al. (2011), Hijaz et al. (2015)
Protein content	0.05–5 µg/µl	α-Amylase, cop9 complex, cullin 1, dihydrofolate reductase, dynamin 1, fibronectin, α-glucosidase, GroEL, inorganic pyrophosphatase, internalin A, katanin p60 subunit A, peroxinectin, rap55 protein, various helicases, various splicing factors, and various transcription factors	Nonexhaustive list of proteins detected in honeydew. A large proportion of the proteins in honeydew is from microbial origin. Protein content dependent on species of honeydew-producing insect.	Sabri et al. (2013), Urbaneja-Bernat et al. (2024)
Salicylic acid	48–88 ng/100 µl/day	NA		Schwartzberg and Tumlinson (2013)
ROS	NA	NA	No direct identity of ROS has been reported yet. Presence is suggested due to the presence of enzymes protecting against ROS.	Urbaneja-Bernat et al. (2024), Zhu et al. (2020)

Honeydew can originate from a wide diversity of honeydew producing insects (aphids, treehoppers, mealybugs, etc.). Each of these insects may carry their own gut microbiota and symbionts, which can alter the diversity in nutrients present in the honeydew (Hansen and Moran 2011, Jossart et al. 2025; see section "Microbial-mediated effects on honeydew chemical composition and its functional consequences" for more details). Additionally, the honeydew can be deposited on different leaf surfaces, each containing its own phylloplane inhabiting bacterial community. As a result, defining a key microbial community of honeydew is not possible. Therefore, we discuss only the bacterial and fungal diversity, which has been detected within honeydew. Although they are discussed separately, both are shaped by the same major ecological pressures: dispersal constraints, chemical stressors, species interactions, and rapid turnover.

### Bacterial diversity of honeydew

The honeydew-inhabiting bacterial community comprises both bacteria derived from the honeydew-producing insects, the phyllosphere, the surrounding environment, and from honeydew consumers (Leroy et al. 2011, Gu et al. 2023, Liu et al. 2024, Jossart et al. 2025, Zhao et al. 2026). However, assigning specific bacterial taxa to these origins remains challenging, as only one study has directly compared the bacterial community in honeydew to the external and internal bacterial community of the honeydew-producing insect (Zhao et al. 2026). Nevertheless, studies characterizing honeydew bacterial community report both facultative endosymbionts (e.g. *Hamiltonella* spp.; Darby and Douglas 2003), and obligate endosymbionts [e.g. *Buchnera* spp. (Glacet et al. 2025), specific strains of *Serratia* spp. (Pons et al. 2019), and *Tremblaya* sp. (Zhao et al. 2026)] from honeydew-producing insects. Although, it should be noted that *Buchnera* spp. and *Hamiltonella* spp. have only been detected in honeydew using 16S rRNA metabarcoding (Darby and Douglas 2003, Glacet et al. 2025). Making it difficult to conclude that these endosymbionts play a role within the microbial community of honeydew. Conversely, both *Serratia* spp. and *Tremblaya* sp. have been identified using both cultivation techniques and RNA sequencing respectively (Pons et al. 2019, Zhao et al. 2026). Indicating that these endosymbionts may contribute to community assembly. However, further research is necessary to elucidate their exact role on the community assembly of honeydew. Further, it has been hypothesized that the deleterious facultative endosymbiont *Ricketsiella* spp. may be horizontally transmitted through insect honeydew, however direct evidence of its presence in honeydew is currently lacking (Gu et al. 2023). In addition to endosymbionts, other gut-associated bacteria such as *Erwinia* [e.g. *E. aphidicola, E. tasmaniensis* (Liu et al. 2024)], *Pantoea* spp. [e.g. *P. cypripedii* (Shamim et al. 2019), *P. dispersa* (Shamim et al. 2019, Wari et al. 2019a)], *Pseudomonas* [e.g. *Pseudomonas* sp. (Zhao et al. 2026)], *Lactobacillus* spp. [e.g. *L. plantarum* (Gustaw et al. 2018, Zhao et al. 2026)], and some nonsymbiotic strains of *Serratia* spp. [*S. symbiotica* (Du et al. 2023)], have been found within insect honeydew.

Beyond gut-associated bacteria, a range of osmotolerant bacteria from diverse origins such as the phyllosphere, wind, and insects feeding on honeydew have been detected within insect honeydew. These include but are not limited to: acetic acid bacteria [e.g. *Acetobacter aceti, A. liquefaciens, A. xylinum*, and *Glucinobacter oxydans* (Ashbolt and Inkerman 1990)], some entomopathogenic bacteria such as *Pseudomonas syringii* (Smee and Hendry 2022), and commensal bacteria such as *Staphylococcus* spp. [e.g. *S. capitis* (Liu et al. 2024), *S. sciuri* (Wari et al. 2019a, Glacet et al. 2025), *S. xylosus* (Wari et al. 2019a), etc.], *Bacillus* spp. [e.g. *Bacillus* sp. (Zhao et al. 2026), *B. cereus* (Liu et al. 2025), *B. safensis* (Liu et al. 2024)], and *Acinetobacter* spp. [e.g. *A. bereziniae* (Liu et al. 2024) and *A. soli* (Glacet et al. 2025)]. It is important to note however, that genus taxonomic affiliation is insufficient to conclude the origins of each taxon.

### Fungal diversity of honeydew

In addition to bacteria, honeydew is commonly colonized by yeasts and true fungi, particularly sooty moulds. Many of these fungi cope with high osmotic pressure either by rapidly adjusting their cell volume (Saldaña et al. 2021) or via the high-osmolarity glycerol pathway (Yaakoub et al. 2021). This pathway may also contribute to tolerance to ROS (Yaakoub et al. 2022), while other species may react to ROS by encoding various extracellular peroxidases, mono- and peroxygenases, and oxidases (Mattila et al. 2022).

Similar to honeydew-inhabiting bacteria, honeydew-inhabiting fungi can originate either from insect symbionts, gut-related microbiota, or other diverse inocula (He et al. 2013, Pringle and Moreau 2017, Vasse et al. 2017, Flessa et al. 2021, Cheng et al. 2024). However, similar to the bacterial diversity of honeydew, knowledge on the origins of honeydew fungal communities remains limited. The three genera *Acremonium* sp., *Fusarium* sp., and *Moesziomyces* sp. of yeast-like symbionts have been identified in plant-hopper honeydew using ITS DNA metabarcoding (Cheng et al. 2024). Additionally, the black-yeast species, *Knufia* spp., also found in the gut of scale insects has been detected in their honeydew (He et al. 2013, Vasse et al. 2017). Sooty moulds are the most ubiquitous, abundant, diverse, and most studied group associated with honeydew. The sooty-mould communities largely overlap with those found on the leaves from which the honeydew-producing insects feed (Ricks and Koide 2019, Flessa et al. 2021), indicating that these sap-feeding insects may be able to acquire these fungi and disperse them to new environments. The sooty moulds found within honeydew are primarily found within the orders Capnodiales, Dothideales, and Pleosporales, and a wide range of genera (Hughes 2003, Dhami et al. 2013, Chomnunti et al. 2014). Because sooty moulds are difficult to culture under laboratory conditions and often occur as mixed communities, species-level identification remains challenging (Chomnunti et al. 2014). Nevertheless, microbial taxonomic composition may differ by geographic region, plant species, and insect species (Dhami et al. 2013, Chomnunti et al. 2014, Flessa et al. 2021).

## Microbial-mediated effects on honeydew chemical composition and its functional consequences

Microorganisms associated with honeydew, both within the honeydew producing insect (e.g. endosymbiont infection status) and microorganisms colonizing it after excretion can substantially alter its chemical composition (Jossart et al. 2025). The extent and trajectory of these changes depend strongly on the initial composition of the honeydew, which is already shaped by microbial processes within the insect (Hansen and Moran 2011, Jossart et al. 2025). The symbiont community within the honeydew-producing insect strongly impacts honeydew chemical composition prior to excretion (Macheda et al. 2026). Proteins such as GroEL excreted by symbionts into the insect gut or haemolymph, can activate plant pattern-triggered immunity, thereby increasing the abundance of secondary metabolites in the phloem and ultimately affecting the honeydew’s chemical composition (Chaudhary et al. 2014, Zhang et al. 2025).

Upon excretion, honeydew chemical composition is largely determined by the identity (van Neerbos et al. 2020) and life-stage of the honeydew-producing hemipteran (Pringle et al. 2014), the host plant and soil chemistry (Pringle et al. 2014, Whitaker et al. 2014). Furthermore, honeydew chemical composition is also determined by the environmental conditions such as temperature, CO_2_ concentration, and relative humidity (Blanchard et al. 2022, Fernández de Bobadilla et al. 2025). Fresh honeydew composition is dominated by carbohydrates, including monosaccharides (i.e. glucose and fructose), disaccharides (i.e. sucrose, trehalose, and maltose), and oligosaccharides (i.e. raffinose, erlose, and melezitose) (Tena et al. 2013, van Neerbos et al. 2020, Urbaneja-Bernat et al. 2024; see Table 1). Besides carbohydrates, honeydew also contains smaller amounts of amino acids, organic acids, secondary metabolites, and ROS derived from both plant sap (e.g. salicylic acid and hydrogen peroxide) (Ewart and Metcalf 1956, Schwartzberg and Tumlinson 2013), Zhu et al. 2020, Urbaneja-Bernat et al. 2024; see Table 1). Ant tending (i.e. the protection and harvesting of honeydew by ants) can further modify both its composition and excretion. For example, when the aphid *Tuberculatus quercicola* was tended by the ant species *Formica yessensis*, Yao and Akimoto (2001) found a higher proportion of sucrose within the aphid honeydew and a lower concentration of glucose. Simultaneously, they found that the ant-tended aphids produced honeydew more frequently but in smaller amounts than their nontended counterparts (Yao and Akimoto 2001). They suggested that this change in the composition was due to a decreased hydrolosis potential within the aphid (Yao and Akimoto 2001). Conversely, Zhou et al. (2015), found no such changes in the sucrose and glucose concentrations within the honeydew composition of mealybugs tended by ants, indicating no decrease in the hydrolosis potential. However, they did report a marked decrease of xylose and increase of melezitose (Zhou et al. 2015). Despite the results reported by Yao and Akimoto (2001) other aphid species have shown an increased melezitose concentration in honeydew in response to ant-tending (Fischer and Shingleton 2001, Vantaux et al. 2011). Further, ant-tending has been shown to also increase the concentration of amino acids within aphid honeydew (Yao and Akimoto 2002). Based off of the absence of reports during the preparation of our review, we found no current hypothesis explaining the mechanism behind the increased melezitose concentration within honeydew. The concentration of melezitose within honeydew from ant-tended aphids differed between clonal lines (Vantaux et al. 2011). Therefore, we hypothesize that either the endosymbiont presence within the insects or the genetic variation of the insects could play a role in the synthesis of melezitose in response to ant tending. However, more research is necessary.

Once honeydew is deposited on the plant surface, it is rapidly colonised by environmental microorganisms that further transform its chemical composition (Lenaerts et al. 2016, Butterworth et al. 2022, Martin et al. 2022). Microbial colonization drives two concurrent processes that reshape honeydew chemistry. First, resource exploitation, in which microbes consume easily accessible substrates (e.g. simple sugars and free amino acids) and convert them into microbial biomass (Görke and Stülke 2008). Second, resource modification, in which microbes introduce new metabolites (e.g. microbial amino acids, alcohols, organic acids, and proteins) into the substrate (Table 2). In addition, microbes within the honeydew are able to secrete polysaccharides which trigger both the jasmonic acid and salicylic acid pathways in the plant, which results in decreased feeding in the honeydew-producing insect (Zhao et al. 2026). These microbial changes result in honeydew not being a relatively static exudate, but rather a dynamic microbially engineered ephemeral resource patch (Lenaerts et al. 2016, Butterworth et al. 2022, Martin et al. 2022). As the chemical environment shifts, it may favour microorganisms that are better adapted to these altered conditions. This in turn may lead to microbial succession that further modifies honeydew chemistry. Similar processes of microbial succession have been observed in other carbohydrate rich substrates, such as several spontaneous food and beverage fermentations (De Roos and De Vuyst 2018, De Vuyst and Leroy 2020), and floral nectar (Fukami 2015, Evans et al. 2016, Chappell et al. 2022). Carbohydrate consumption and fermentation by microbes also alters the VOC profile of honeydew (Leroy et al. 2011, Liu et al. 2024, Glacet et al. 2025). Specifically, the relative abundance of acetals decreases while microbially derived compounds such as alcohols, benzene derivatives, organic acids, and terpenes increase (Liu et al. 2024, Glacet et al. 2025). Such shifts in VOC profiles have been shown to alter insect foraging behaviour, leading to increased attraction of predators and parasitoids of honeydew-producing insects (Leroy et al. 2011, Liu et al. 2024, 2025, Schulz-Bohm et al. 2017). Also, microbial consumption of the easily fermentable sugars in honeydew (i.e. mono and disaccharides) increases the relative abundance of oligosaccharides such as erlose, melezitose, and trehalose and may change the quality of honeydew for other consumers.

**Table 2:** Consequences of microbial metabolism for the chemical composition of hemipteran honeydew.

Component	Microbial action	Consequence for honeydew chemistry	Functional consequence	References
Carbohydrates	Depletion of mono- and disaccharides (e.g. sucrose, glucose, and fructose); fermentation of sugars into alcohols and organic acids	Decrease in mono- and disaccharides. Increased relative abundance of oligosaccharides (e.g. erlose, melezitose, and trehalose).	Decreased nutritional value for mutualists and natural enemies.	Geerinck et al. (2025), Goelen et al. (2018), Nelson and Mooney (2022)
Proteins	Production of proteins as part of the microbial metabolism	Introduction of microbial proteins (e.g. GroEL) and enzymes into the honeydew	Induced plant-defence responses, results in increased abundance of plant-secondary metabolites in honeydew	Chaudhary et al. (2014), Sabri et al. (2013), Urbaneja-Bernat et al. (2024)
Amino acids	Consumption of amino acids; *de novo* synthesis of new amino acids due to microbial metabolism	Shifts from plant- and insect derived towards microbially derived amino acids	None have been reported	Pan et al. (2022), Sasaki et al. (1990)
VOCs	Microbial fermentation drives the production of VOCs, including alcohols, ketones, benzene derives, and terpenes	Increased presence of alcohols, and microbial fermentation products	Increased attraction of predators and parasitoids of honeydew-producing insects. Increased attraction of honeydew consumers	Glacet et al. (2025), Hung et al. (2015), Leroy et al. (2011), Liu et al. (2024, 2025), Schulz-Bohm et al. (2017)

## Chemical-mediated effects in honeydew microbial dynamics

**Figure 1.**
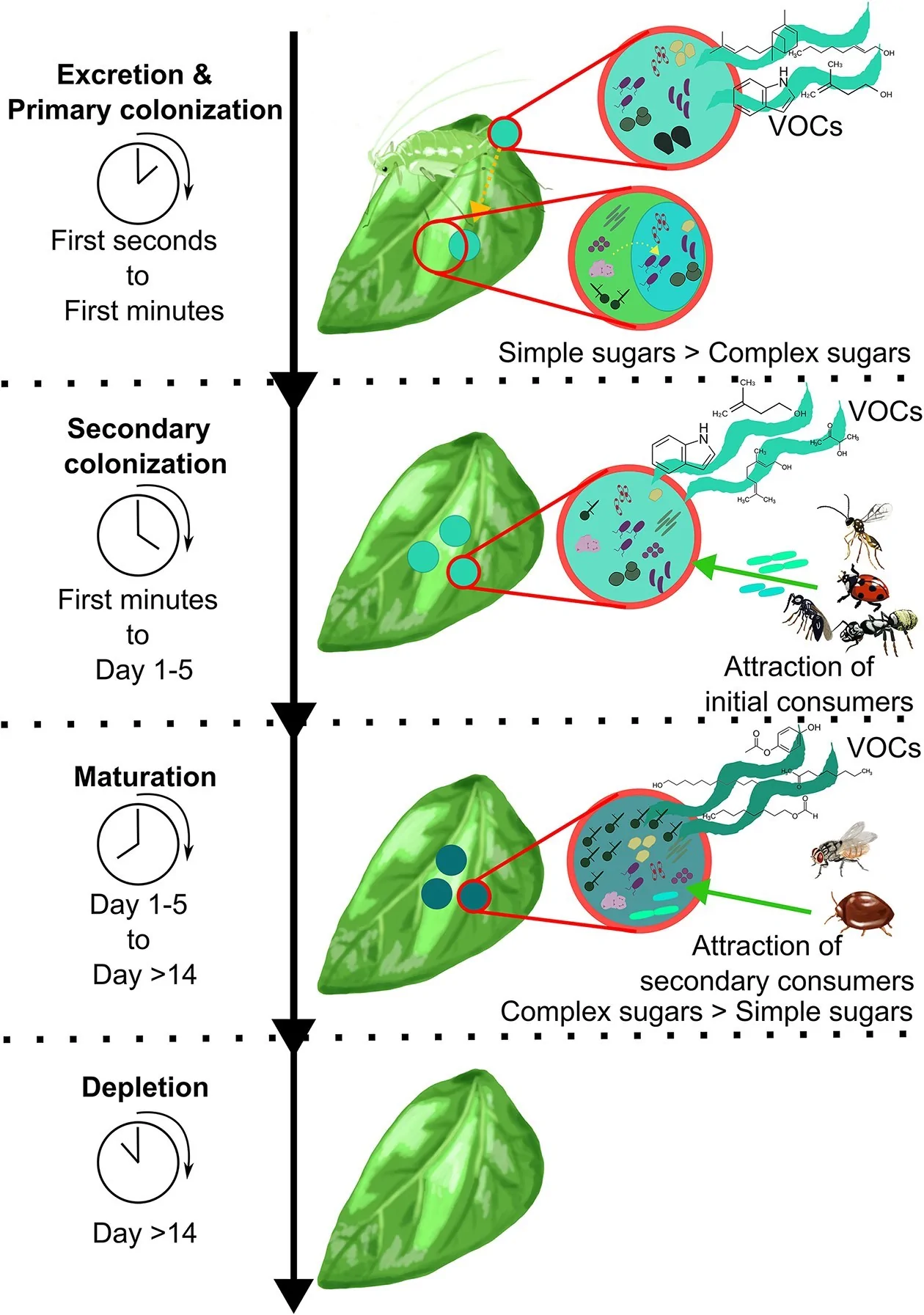
Hypothetical schematic overview of the successional microbial dynamics in insect honeydew. The first stage is the excretion and primary colonization of honeydew (1), in which a hemipteran insect (here represented by an aphid) excretes honeydew, containing both symbionts and other gut-associated microbes. If the honeydew is not harvested before deposition on the leaf surface, it is colonized by the phyllosphere-inhabiting microbes. The potential volatiles emitted within this stage are similar to those emitted by fresh honeydew (In this figure represented by (E)-non-2-en-1-ol, indole, isoprenol, and trans-α-bergamotene). Further, at this stage the honeydew has the highest ratio of simple sugars (e.g. glucose, fructose, etc.) in comparison to more complex oligosaccharides (e.g. erlose, raffinose, etc.). This initial stage is the shortest stage and can last anywhere from seconds to minutes. After phyllosphere-inhabiting microbes have colonized honeydew, the volatile profile changes, and primarily consists of bacterial volatiles which attract honeydew consumers. (e.g. insect natural enemies and hyperparasitoids). These initial consumers further introduce microbes within the honeydew combined with microbes carried by the wind representing the secondary colonization stage (2). This stage can last anywhere from minutes to 1–5 days. During the first two stages, the bacteria and yeasts primarily metabolize and ferment the simple sugars (i.e. mono- and disaccharides) (Görke and Stülke 2008). This consumption results in a ratio shift from simple sugars to complex sugars (i.e. oligosaccharides), as well as an increased production of bacterial volatiles (In this figure represented by acetoin, citronellal, indole, and isoprenol). As sooty moulds are more efficient consumers of complex sugars than bacteria and yeasts, the microbial community will become dominated by these species. This stage represents the maturation stage of honeydew (3). The volatile profile shifts from bacterial volatiles to more fungal volatiles (In this figure represented by 1-dodecanol, 4-hydroxyphenyl acetate, 2-nonanone, and octyl formate), changing the community of consumers towards those attracted to fungal volatiles [referred to as secondary consumers (e.g. house flies and beetles)]. This maturation stage represents the longest time period from days to weeks. Finally, these consumers together with the fungi are expected to fully consume the honeydew which results in its terminal stage. The terminal stage here is referred to as the depletion stage (4). This marks the stage where all of the sugars and resources are consumed and are no longer present in the environment.

As discussed previously, honeydew is a microbially engineered ephemeral resource patch subject to changes over time. These microbially induced chemical changes, in turn, alter honeydew microbial community, leading to microbial succession (Glacet et al. 2025) and further shifts in the honeydew chemistry. Similar processes in microbially induced chemical changes have been observed in other systems, including spontaneous food and beverage fermentations (De Roos and De Vuyst 2018, De Vuyst and Leroy 2020), and within the microbial community within floral nectar (Fukami et al. 2015, Evans et al. 2016, Chappell et al. 2022). Within both systems, the initial state is marked by a high concentration of carbohydrates (De Roos and De Vuyst 2018, De Vuyst and Leroy 2020, Álvarez-Pérez et al. 2024). Further, due to either microbe–microbe interactions or priority effects, the composition of these substrates can change with time, shifting away from largely substrate (or plant-related) metabolites towards microbe related metabolites (De Roos and De Vuyst 2018, De Vuyst and Leroy 2020, Chappell et al. 2025). Therefore, by analogy with these processes, we propose four stages, which we hypothesize are distinct and define the successional dynamics of the honeydew microbiome: (1) honeydew excretion and primary microbial colonization, (2) secondary microbial colonization, (3) honeydew maturation during which fungal growth dominates, and (4) depletion (Fig. 1). Each stage is likely characterized by a unique microbial diversity, nutrient composition, and ecological function. The total time period over which these stages occur is still unknown, but it can be assumed that they last from a few hours to several weeks depending on temperature, humidity, and honeydew consumers.

### Stage 1. Excretion and primary colonization

Freshly excreted honeydew contains a high concentration of mono- and disaccharides, and a lower concentration of oligosaccharides (Wäckers et al. 2006, van Neerbos et al. 2020). We hypothesize that at this stage, the honeydew microbiome is likely dominated by endosymbionts and other gut microbes originating from the honeydew-producing insect (e.g. *Acremonium* sp., *Erwinia* spp., *Pantoea* spp., *Pseudomonas* spp., *Serratia* spp., *Tremblaya* sp. etc.) (Smee and Hendry 2022, Cheng et al. 2024, Glacet et al. 2025, Zhao et al. 2026). Once the honeydew lands on the leaf surface, phyllosphere inhabiting microbes such as *Acinetobacter* spp., *Bacillus* spp., *Staphylococcus* spp., sooty moulds, etc. are likely the first ones to colonize honeydew (Flessa et al. 2021, Li et al. 2025, Liu et al. 2025, Zhao et al. 2026). As such, this community assembly stage is largely driven by the identity and life stage of the insect as well as symbiont presence within the insect. The volatiles emitted in this stage resemble that of freshly excreted honeydew, and thus contain more acetals, and some benzene derived compounds (Glacet et al. 2025). It has been reported that aphids and other Hemipterans produce a honeydew droplet every 15–40 min (Douglas 2009). Further, a recent study reported the honeydew ejaculation velocity of the Spotted lantern fly (*Lycorma delicatula*) to be 1.64 m/s (Ha et al. 2026). Based off of these reports, the excretion of honeydew and the subsequent primary colonization of the phyllosphere inhabiting microbes likely occurs quite rapidly. As a result, we hypothesize this stage to be the shortest of the four, lasting anywhere from mere seconds to minutes. This stage is likely the shortest of the four, lasting anywhere from mere seconds to minutes. If the honeydew is harvested by ants or other honeydew consumers, the subsequent stages do not occur (Nelson and Mooney 2022). In fact, many honeydew droplets may even never progress beyond this point. This termination of the subsequent stages results from the consumption of the honeydew before it enters the environment.

### Stage 2. Secondary colonization

Additional microbes from various sources may be introduced into the honeydew after it has touched the leaf surface. Specifically, we hypothesize that this stage happens after the underlying phyllosphere has further colonized the honeydew during the first stage (Smee and Hendry 2022, Li et al. 2025, Liu et al. 2025). These additional microbes may originate from the honeydew consumers, i.e. insects and vertebrates [e.g. birds (Nascimento de Mattos et al. 2025) or geckos (Fölling et al. 2001)], as well as from the wind and rain. We predict that at this stage the microbial community is primarily dominated by bacteria. This would result in the honeydew volatilome being more dominated by bacterial volatiles [e.g. alcohols, ketones, alkenes, and terpene derived compounds (Glacet et al. 2025)]. The honeydew consumers attracted to the honeydew will be those primarily attracted to bacterial volatiles such as insect natural enemies (Leroy et al. 2011, Liu et al. 2024, Li et al. 2025), and hyperparasitoids (van Neerbos et al. 2023a). These events are likely to introduce novel microbial taxa, and mark the onset of stochastic and environment-dependent community assembly. In this stage, microbial composition is mainly driven by microbial dispersal limitation (Evans et al. 2016), filtering of the microbial community due to environmental changes within the honeydew (Yan et al. 2019), and intermicrobial species interactions (Gralka et al. 2020). These intermicrobial species interactions, include priority effects, which shape downstream succession (Fukami 2015, Evans et al. 2016). We predict that the microbes thriving in this phase are those from stage 1, as well as microbes vectored into the honeydew that are able to survive its extreme conditions (e.g. acetic acid bacteria, lactic acid bacteria, etc.). The bacteria and yeasts in this stage primarily metabolize and ferment the available simple sugars (Görke and Stülke 2008). Finally, most likely sooty mould spores can also be found within this stage, as they may be carried through the wind, scavenging insects, or from the leaf surface, although this needs to be verified.

### Stage 3. Maturation

As microbial metabolism progresses, nutrient depletion and microbial metabolic accumulation is likely to differentially impact the growth of bacteria and sooty moulds. During this stage, most of the mono- and disaccharides are likely consumed by the bacterial and yeast community in the honeydew, increasing the proportion of oligosaccharides (Görke and Stülke 2008). While some bacterial clades metabolize oligosaccharides, due to the high energetic cost of this process, most only metabolize mono- and disaccharides (Görke and Stülke 2008). Sooty moulds grow epiphytically and are adapted to the unique physiological characteristics of honeydew in a diverse pH range and high ROS and oligosaccharides content (Table 1; Dhami et al. 2013, Lv et al. 2023). Further, some sooty moulds such as the clade *Capnodiales* have been reported to metabolize oligosaccharides (Abdollahzadeh et al. 2020). Additionally, research on coconut and banana leaves infested with both honeydew-producing insects and sooty moulds, show an increase in honeydew metabolites and a lower nutrient quality (Hölscher et al. 2017, Arun et al. 2021). The degradation of oligosaccharides by sooty moulds, allow for a faster growth rate of sooty moulds compared to bacterial or yeast clades (Abdollahzadeh et al. 2020, Arun et al. 2021). Thus, as honeydew is exposed for a longer time to the environment, there is a community turnover from bacterial dominance to sooty mould dominance. At this stage, the honeydew volatile profile changes to such an extent that attraction of several macro-consumers is reduced (Glacet et al. 2025). In fact, there is currently no evidence of aphid predators or (hyper-)parasitoids being attracted to the sooty moulds within honeydew. However, the sooty moulds can be attractive to more detritivorous insects such as sooty mould feeding beetles (Josephrajkumar et al. 2018) and house flies (Hung et al. 2015).

### Stage 4. Depletion

In this final stage, sooty moulds and the secondary consumers fully deplete the honeydew resource patch limiting further microbial growth. This terminal stage represents an endpoint in the existence of a honeydew patch in the ecosystem.

Research on the successional dynamics within insect honeydew is still limited. However, insights from the microbial ecology in similar sugar-rich ephemeral environments like floral nectar or rotting fruits may provide a conceptual framework on honeydew microbial dynamics. Specifically, priority effects, where early arriving microbes rapidly alter chemistry and resource availability and thereby influence which microbes can establish later. These priority effects may play an important role in shaping subsequent microbial community assembly within insect honeydew (Fukami 2015, Chappell et al. 2022, Glacet et al. 2025). Given that the initial honeydew microbiome may be dominated by some insect endosymbionts, gut microbes and phyllosphere-inhabiting microbes (Gu et al. 2023, Cheng et al. 2024, Glacet et al. 2025, Zhao et al. 2026), priority effects can arise from the interactions of multiple species. Nevertheless, given that sooty moulds typically dominate late-stage honeydew communities (Chomnunti et al. 2014, Arun et al. 2021), the metabolic activities of the early colonizers are likely the engine driving the successional transition towards a sooty-mould dominated community. We hypothesize that honeydew-inhabiting microbes may overcome the loss of their habitat by being vectored to new environments. The hoverfly *Episyrphus balteatus*, which feeds on aphids and honeydew, can harbour microbes commonly found in both the aphid and its honeydew (e.g. *S. symbiotica, B. aphidicola*, and *Acinetobacter* sp.) (Chang et al. 2024). This suggests that honeydew consumers can also acquire and transmit honeydew-inhabiting microbes to novel environments. However, further research should be performed if the honeydew-inhabiting microbes rely on honeydew consumers to be vectored to new environments, or if the microbes have alternative methods to survive the habitat loss.

## Honeydew microbial volatiles mediate interspecific interactions

As a metabolic by-product of herbivores, honeydew VOCs serve as an important cue signalling herbivore presence to their natural enemies (Fernández de Bobadilla et al. 2024). Increasing evidence suggests that microorganisms within honeydew mediate multitrophic interactions through the production of microbial VOCs (Leroy et al. 2011, Schulz-Bohm et al. 2017, Liu et al. 2024, 2025). While the honeydew microbiome is diverse, only a subset of microbial taxa contributes to the production of behaviourally active chemical cues that attract higher trophic level insects (Leroy et al. 2011, Liu et al. 2025, Zhang et al. 2023). For example, only three bacteria (*Brachybacterium* sp., *Kocuria* sp., and *Microbacterium* sp.) from mealybug honeydew are directly responsible for the volatile profile attractive for fire ants (Zhang et al. 2023). Moreover, different natural enemies may respond differently to microbially derived olfactory signals (Li et al. 2025, Liu et al. 2025). For instance, when the ladybeetle *Propylea japonica* was offered the VOC profile of either *Acinetobacter* sp. or *Pseudomonas* sp. from the honeydew of *Aphis gossypii*, it displayed a stronger attractive olfactory response, compared to the crude honeydew (Li et al. 2025). Meanwhile, Liu et al. (2025) analysed the microbial VOCs from *A. gossypii* honeydew, which attracted the ladybeetle *Hippodamia variegata*. They found that a consortium of *Acinetobacter* sp., *Bacillus cereus, Enterobacter* spp., and *Leclercia adecarboxylata* were responsible for the production of VOCs attractive for *H. variegata* (Liu et al. 2025). Interestingly, the honeydew tested in both studies came from the same source of *A. gossypii* reared on healthy cotton leaves (Li et al. 2025, Liu et al. 2025). Both studies identified both *Acinetobacter* sp., and *Enterobacter* spp. within the honeydew of *A. gossypii* honeydew (Li et al. 2025, Liu et al. 2025). The production of the honeydew volatiles was found to primarily originate from *Acinetobacter* sp. and *Enterobacter* spp. (Liu et al. 2025). However, while the microbial communities were roughly similar, the volatiles found to be attractive for the two ladybeetle species were different (Li et al. 2025, Liu et al. 2025).

Evidence suggests that the ecological impact of microbial VOCs on interspecific interactions is driven by individual compounds rather than by the full VOC profile of the key signallers (Goelen et al. 2021, van Neerbos et al. 2023a, Li et al. 2025, Liu et al. 2024, 2025). These individual VOCs can be as or even more attractive, to natural enemies than the full mixture of compounds (Goelen et al. 2021, Liu et al. 2024, 2025, Li et al. 2025). For example, Liu et al. (2024) examined the attractiveness of microbial volatiles from aphid honeydew and identified the bacterial species *Lysinibacillus fusiformis* as the most attractive bacterium for the parasitoid wasp *Aphidius gifuensis*. They further demonstrated that a simplified blend of two compounds from the VOCs of this bacterium 1-ethyl-2-methylbenzene and 2-butyl-1-octanol, elicited higher parasitism rates of *A. gifuensis* than the complete volatilome (Liu et al. 2024).

The VOC blend emitted by the honeydew-inhabiting microbes also change in time (Glacet et al. 2025). These temporal changes in VOC profiles correspond to transitions in our proposed successional framework (Fig. 1). These temporal shifts may alter the window in which natural enemies are attracted to honeydew. Furthermore, the interaction between insect endosymbionts found within honeydew and their excreted proteins (e.g. GroEL) trigger plants immune responses, resulting in the emission of a different blend of plant VOCs (Chaudhary et al. 2014). Additionally, honeydew-inhabiting microbes trigger plant defence responses, which limit both the feeding and growth of honeydew-producing insects (Zhao et al. 2026). Some studies suggest that proteins secreted by other honeydew-inhabiting microbes also induce plant resistance to pest insects or activate pattern-triggered immunity, which in turn leads to the emission of a volatile profile similar to herbivore-induced plant volatiles (HIPVs), attracting insect natural enemies (Wari et al. 2019a, b, Zhang et al. 2025).

Additionally, there is increasing evidence that microorganisms colonizing honeydew, play an important role as plant bodyguards by directly killing honeydew-producing insects (Smee and Hendry 2022, Berings et al. 2026). Specifically, epiphytic entomopathogenic microorganisms, such as *Pseudomonas syringae*, benefit strongly from the nutrients in the honeydew, thereby increasing their cell numbers, leading to increased insect mortality rates (Smee and Hendry 2022). The increased mortality rate of the honeydew-producing insects results in the resource patch no longer being replenished. As a result, the honeydew patch can eventually cease to exist, which results in a habitat loss for the honeydew-inhabiting microbes. This habitat loss could result in a lower growth rate for epiphytic microorganisms such as *P. syringae* (Smee and Hendry 2022). However, for nonepiphytic honeydew inhabiting microorganisms, this habitat loss could potentially be mitigated by attracting insect consumers on or in which they are able to be vectored to new environments. However, currently no direct evidence has been published of insect consumers vectoring honeydew microbes to new honeydew patches. Nevertheless, Chang et al. (2024) did find a horizontal transfer of *S. symbiotica* from the aphid prey to the hoverfly *E. balteatus* through 16S rRNA sequencing. They further hypothesized that this symbiont may have been transferred to *E. balteatus* through the environment (Chang et al. 2024). This may hint that some microbes may be dispersed by insects antagonistic for honeydew production. Previous research on microbial ecology has shown that a low cell count is usually sufficient for colony formation (Álvarez-Pérez et al. 2019). Therefore, honeydew-inhabiting microbes may only be dispersed to new environments through their adhesion capability to their potential vectors. Research on floral nectar and the western flower thrips *Frankliniella occidentalis* feeding on floral nectar, has shown that the dispersal ability of the microbes partially depends on their adhesion ability (Vannette et al. 2021). This adhesion ability may be a separate trait (e.g. biofilm production) from the microbes ability to modify the olfactory behavior of the insect consumers. Although more research into honeydew inhabiting microbes is necessary.

## Unlocking the potential of honeydew microbial ecology

We have previously discussed how honeydew-inhabiting microorganisms alter honeydew chemical composition and as a result mediate interspecific interactions. Here, we discuss how these changes can affect ecosystem functioning and how they can be exploited to improve biological control.

### Ecosystem functioning

Microbial conversion of honeydew alters both honeydew chemistry and its volatile profile. These microbial-induced shifts in honeydew quality and detectability influence the duration and strength of interactions among honeydew-feeding organisms thereby modifying multitrophic interactions (Hogervorst et al. 2008, Tena et al. 2016, van Neerbos et al. 2020, Nelson and Mooney 2022). For example, honeydew and honeydew-inhabiting microbes mediate the behaviour of tending ants, which in turn restructure entire arthropod assemblages (Styrsky and Eubanks 2007). Specifically, plants containing honeydew producing Hemipterans and are tended by ants, show a decrease in plant damage (Styrsky and Eubanks 2007). Endosymbionts of honeydew-producing insects alter ant behaviour by changing the amount of honeydew being produced (Macheda et al. 2026) as well as the presence of cuticular hydrocarbon profiles of the endosymbiont insect host (Oliver et al. 2010, Mandrioli et al. 2016, Hertaeg et al. 2021). Ants can strongly reduce predator and parasitoid abundance while simultaneously suppressing nontended herbivores (Foronda et al. 2026). This could result in less insect consumers introducing novel taxa into the honeydew. In turn, this could modify the intensity of biological control, intraguild interactions, and the local richness of herbivores and natural enemies (Styrsky and Eubanks 2007, Plata et al. 2025). These ecosystem-scale effects are likely structured by the successional stages of the honeydew microbiome, with shifts in community composition driving distinct nutrient profiles and volatile blends over time.

Importantly, as discussed in more detail in the section "Microbial-mediated effects on honeydew chemical composition and its functional consequences", microbial induced changes in honeydew also affect plant fitness. Honeydew microbes alter phyllosphere microbial communities (Stadler and Müller 1996, Sohrabi et al. 2023, Wolfgang et al. 2023) and prime plant defences, which in turn can result in lower herbivore performance (Zhao et al. 2026). Additionally, honeydew deposition triggers changes in soil biota that cascade back to plant growth. In a field experiment, honeydew significantly increased soil microbial biomass and respiration, boosted microfauna and earthworm biomass, altered litter mineralisation kinetics, and shifted mesofauna composition (Milcu et al. 2015). These changes were accompanied by marked shifts in plant resource allocation, such as increased shoot: root ratios and altered crown architecture showing that honeydew-induced microbial responses in the soil can scale up to change canopy structure (Milcu et al. 2015). The introduction of carbon from honeydew also alters the soil C:N balance (Seeger and Filser 2008), thereby modulating nutrient cycling and linking canopy-microbial processes with belowground decomposition. Thus, honeydew-inhabiting microbes are crucial connectors linking the microbiota and its resources in the phyllosphere, canopy, and soil.

### Biological control

Honeydew mediates multitrophic interactions, which affect biological control because honeydew is simultaneously one of the most abundant carbohydrate sources in agricultural systems and a major source of volatiles attracting natural enemies (Tena et al. 2016, Fernández de Bobadilla et al. 2024). Many honeydew producers are key pests, yet the honeydew they excrete feeds a diverse community of beneficial insects, including parasitoids, predators, and pollinators, that depend on carbohydrate-rich foods to maximise longevity, fecundity, and foraging activity. Honeydew may be one of the most underutilized ecological resources in pest management, harbouring microorganisms that produce bioactive volatiles and proteins with direct and indirect biocontrol potential. There is increasing evidence that microorganisms that colonize and exploit honeydew as a food source play an important role in crop protection by directly killing honeydew-producing insects (Nadarasah and Stavrinides 2011, Smee and Hendry 2022, Berings et al. 2026). However, it should be noted that the extent to which this entomopathogenic potential translates into meaningful crop protection under field conditions remains unknown. Furthermore, honeydew microbes can indirectly shape biological control outcomes by altering the feeding behaviour of both pests (Zhao et al. 2026) and their natural enemies through the emission of VOCs (Álvarez-Pérez et al. 2024, Leroy et al. 2011, Li et al. 2025, Liu et al. 2024, 2025). These microbe-derived volatiles act as potent VOCs that mediate attraction or avoidance responses of natural enemies of pests. Therefore, these microbial VOCs have considerable potential for the development of targeted attractants or repellents that can be used in pest management programs. Importantly, as discussed in the section "Honeydew microbial volatiles mediate interspecific interactions", individual synthetic VOCs or simplified blends often outperform complete honeydew-derived bouquets, making optimisation of specific compound combinations a more feasible strategy than deploying full scent profiles (Goelen et al. 2021, Li et al. 2025, van Neerbos et al. 2023b). For example, this technique was used to develop a blend of styrene and benzaldehyde (synthetic bacterial VOCs) to attract the aphid parasitoid *A. colemani* over a distance of 5 m in a field-experiment (van Neerbos et al. 2023b).

**Figure 2.**
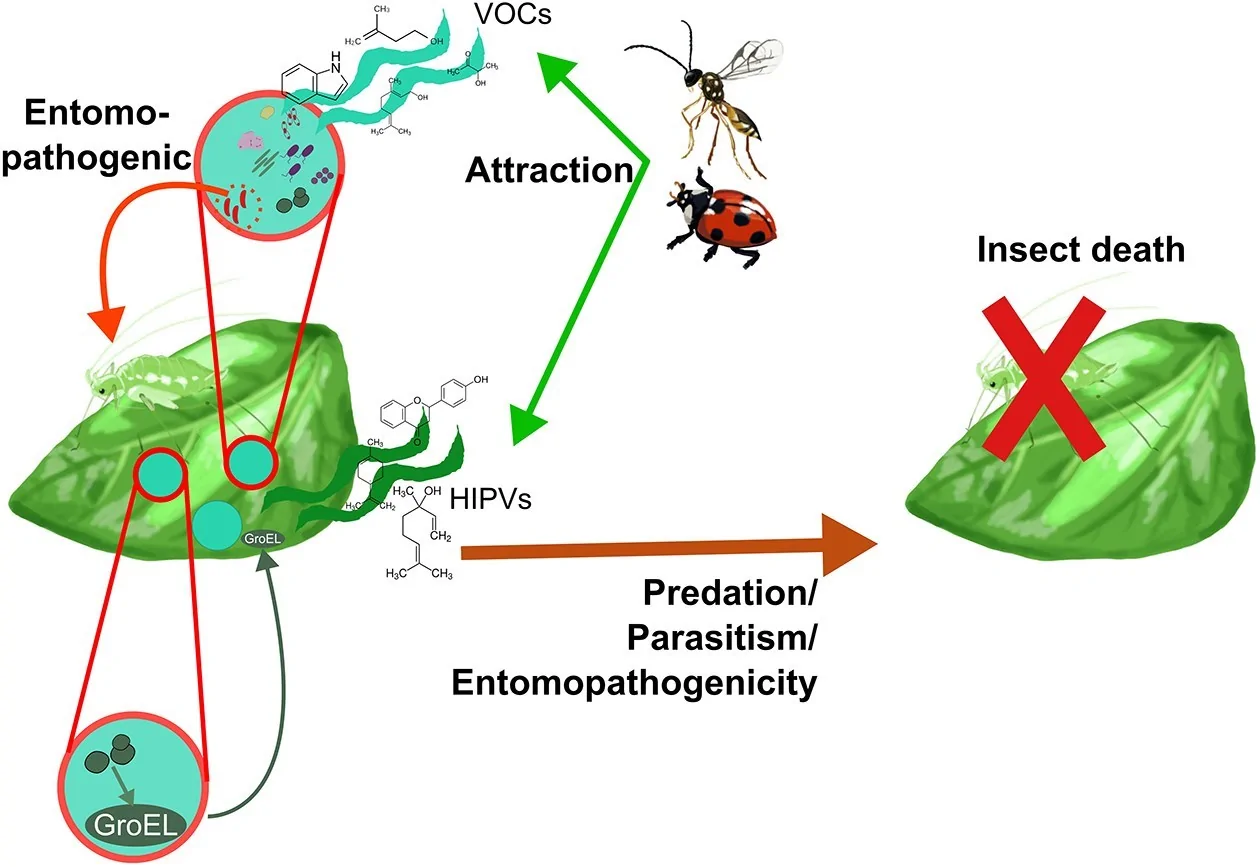
Schematic overview of how honeydew-inhabiting microorganisms can aide in successful biological control of herbivorous honeydew-producing insects. Some honeydew-inhabiting microorganisms show entomopathogenicity in honeydew leading to direct insect mortality. Further, honeydew-inhabiting microorganisms excrete VOCs (In this figure represented by: acetoin, citronellal, indole, and isoprenol), attracting natural enemies. Additionally, other microorganisms within the honeydew may excrete proteins such as GroEL, which act as plant defence elicitors, leading to an increased emission of HIPVs. This may further increase attraction of the natural enemies.

Beyond volatile signalling, some honeydew-associated microbes secrete proteins which act as defence elicitors, activating direct plant defences or promoting natural enemy recruitment (Chaudhary et al. 2014, Wari et al. 2019a, b, Zhu et al. 2020). Further, honeydew-associated microbes are also able to induce plant defence responses directly which results in a lower feeding rate and body mass of the honeydew-producing insects (Zhao et al. 2026). Although this dual mechanism remains largely unexplored, it highlights the broader potential of honeydew microbial ecology to provide novel, multifunctional tools for biological control. Fully integrating honeydew microorganisms and their volatiles into pest management will require a better understanding of how microbial community assembly and environmental context influence both the production of bioactive and sometimes entomopathogenic metabolites and the resulting multitrophic interactions (Fig. 2) (Fernández de Bobadilla et al. 2024).

## Conclusions and future directions

Honeydew is a microbially engineered ephemeral resource patch, which changes over time and space and reshapes ecological interactions. These interactions are primarily mediated through the volatilome of the honeydew, which in turn is primarily shaped by its microbial community. While the microbial community is hard to define, previous studies have consistently identified the bacterial community to consist of: *Acinetobacter* spp., *Bacillus* spp., *Erwinia* spp., *Pantoea* spp., *Pseudomonas* spp., *Lactobacillus* spp., *Serratia* spp., and *Staphylococcus* spp. Conversely, while the true fungal community of honeydew may be more diverse, only sooty moulds have been consistently detected within honeydew. These microbial members within honeydew convert the available nutrients and excrete VOCs and other metabolites. These changes may result in honeydew-mediated ecological interactions to change over time due to microbial succession, which likely follows clear distinct stages. Based on the initial nutritional composition of honeydew, primarily consisting of mono- and disaccharides, the early stages are dominated by bacterial taxa. As these taxa more readily consume the mono- and disaccharides, while not consuming the tri- and polysaccharides at the same rate, the relative ratio would shift towards tri- and polysaccharides. As fungal taxa more readily metabolize these tri- and polysaccharides, later stages of honeydew are more likely dominated by fungi rather than bacteria. This shift from bacteria to fungi also come with a shift from bacterial volatiles to fungal volatiles. In turn this shift in volatiles can result in altered interactions with not only mutualists, such as ants, but also with natural enemies, and insect consumers of honeydew. The changes in honeydew composition in turn could also be exploited in biological control, however more research is necessary before this may be adopted. Honeydew-mediated ecological interactions change over time due to microbial succession that likely follows clear distinct stages. Here, we both synthesized current existing literature on the microbial ecology of honeydew, but also proposed new hypotheses to help the field move forward. Specifically, we identified five important knowledge gaps which future studies should address:

1. Temporal dynamics of honeydew: Most studies have considered the honeydew microbiome to be static over time. Consequently, it remains largely unknown how the overall microbiome (including both bacteria and fungi) evolves over time. We have proposed a theoretical four-stage successional model spanning the lifespan of honeydew, along with the potential mechanisms underlying these dynamics. Experimental validation of this model will help clarify whether honeydew undergoes predictable successional states, and the ecological consequences of each stage.
2. Diversity of the honeydew origin: Most studies on the microbial diversity of honeydew have focused on aphid honeydew (Leroy et al. 2011, Liu et al. 2024, 2025, Glacet et al. 2025, Li et al. 2025), whereas honeydew produced by other hemipterans remains understudied (but see: Wari et al. 2019a, b, Fand et al. 2020). Future studies should therefore include a broader range of Hemiptera to allow more general conclusions.
3. Methodological expansion: Most studies have only focused on exploring the fungal microbiome of honeydew through culturable methods. Recent advances in whole-microbiome analyses will help disentangle factors shaping the honeydew microbiome. Further, microbial interactions within honeydew are largely underexplored. Integrative approaches including manipulative experiments (through synthetic communities), genomics, transcriptomics, and metabolomics will help uncover how microbial community assembly shape honeydew’s interactions.
4. Dispersal mechanisms. Honeydew-inhabiting microbes emit a suite of volatiles that attract honeydew consumers, whose feeding results in the eventual depletion of the microbes’ habitat. Several of these microbes have been found in honeydew-consuming insects but it remains unknown whether they are acquired through honeydew consumption or via interactions with the environment. Future studies should therefore investigate whether honeydew-consuming insects act as vectors, acquiring and dispersing microbes them to new honeydew or other sugar-rich patches. In addition, it should be investigated if this dispersal is linked to any potential trade-offs for microbes.
5. Applications for sustainable agriculture. Decoding the microbial determinants of honeydew’s VOCs could offer innovative sustainable tools for targeted pest suppression, plant defence priming, or beneficial insect recruitment.
